# The willingness of informal caregivers to assist their care‐recipient to use Home Medicines Review

**DOI:** 10.1111/hex.12092

**Published:** 2013-06-06

**Authors:** Stephen R. Carter, Rebekah Moles, Lesley White, Timothy F. Chen

**Affiliations:** ^1^ Faculty of PharmacyThe University of SydneySydneyNSWAustralia; ^2^ Faculty of BusinessCharles Sturt UniversityBathurstNSWAustralia

**Keywords:** caregivers, community pharmacy services, hassles, information‐seeking behaviour, life stress, models, psychological

## Abstract

**Objectives:**

Informal caregivers experience daily hassles – a form of persistent stress, as a consequence of caregiving. This study aimed to develop and test a new theoretical model of health information‐seeking behaviour, the Knowledge Hassles Information Seeking Model (KHISM). KHISM hypothesized that the *knowledge hassles* of caregivers – daily stressors experienced while dealing with tasks which require knowledge about the safety and effectiveness of the care‐recipients' medicines – would influence caregivers' willingness to assist their care‐recipient to use an Australian medication management service, Home Medicines Review (HMR).

**Methods:**

A cross‐sectional postal survey was conducted among 2350 members of Carers (NSW, Australia). Respondents were included in the study if they were involved in medication‐related tasks for their care‐recipient and were not paid as caregivers. Also, their care‐recipient needed to be taking more than five medicines daily or more than 12 doses daily and had not yet experienced HMR. Structural equation modelling was used to test the model.

**Results:**

A total of 324 useable surveys were returned yielding a response rate of 14%. Respondents were quite willing to assist their care‐recipient to use HMR (willingness). The model predicted 51% of the variation in willingness. Knowledge hassles increased positive outcome expectancy (β = 0.40, *P* < 0.05) and indirectly increased willingness.

**Conclusions:**

The more caregivers experience hassles with medication knowledge, the more they perceive HMR to be a helpful information source and the more willing they are to use it. Targeted marketing centred on HMR as an information source may increase caregivers' demand for HMR. Further exploration of the phenomenon of knowledge hassles is warranted.

## Background

In many developed nations, throughout Europe,^1^, ^2^ North America^3^ and in Australia,^4^ the population is ageing and the burden of chronic disease and comorbidity is growing. The burden of disease and associated stress is often shared with informal caregivers. For the purpose of this study, informal caregivers (caregivers) are those persons who care for a person who uses multiple medicines and is not paid to do so. They are very likely to be family members. One of the key reasons for reduced caregivers' quality of life and poor coping strategies is a lack of knowledge about the duties expected of them.^5^ One duty that caregivers lack knowledge about pertains to the specific knowledge required to manage the care‐recipient's medicines.^6^ Medication regimens have become increasingly complex,^7^ as multiple medicines are often used to manage common chronic diseases of ageing.^8^ The more complex a care‐recipient's medication regimen, the more stress a caregiver experiences.^9^ Better caregiver access to medication information may lower their stress and could help them avoid medication problems.^10^ Problems related to the use of medicines are a significant cause of morbidity within Australia.^11^ It is estimated that they result in 2–3% of all hospital admissions, 50% of which may be preventable.^12^

This study deals with caregivers' perceptions of Home Medicines Review (HMR),^13^ a medication safety intervention which aims to resolve medication‐related problems and improve health outcomes for those at greatest risk of medication‐related problems.^14^, ^15^, ^16^ HMR also aims to increase patient and caregiver knowledge of medicines. HMR is provided collaboratively by general practitioners (GPs) and pharmacists. An HMR is initiated with a request from the patient's GP to a pharmacist, who may be their preferred community pharmacist or a consultant pharmacist who works independently. Pharmacists who perform HMR must be accredited by an approved credentialing body.^13^ The pharmacist generally visits the patient and caregiver(s) at their home, for an extended interview regarding medication management issues. Following the visit, the pharmacist sends a written report documenting medication review findings and recommendations to the GP, who then formulates a revised medication management plan with the patient.

Caregivers (whose care‐recipients have received the service) report that the medication information they themselves receive during the HMR service is useful and helps to relieve their emotional stress resulting from dealing with medication issues.^17^ Even though this program increases patients' use of appropriate and evidence‐based therapies,^18^, ^19^, ^20^ evaluation commissioned by the Australian government reported that there had been a suboptimal uptake of HMR, particularly among patients at greatest risk including those with dementia.^21^ The report suggested that caregivers have a key role in enhancing participation and suggested that both patients and their caregivers need to be better informed of the benefits.^21^ The present study deals with the factors that may influence caregivers' willingness to participate in the processes of HMR (for their care‐recipient). Willingness to participate is therefore defined as the willingness of a caregiver to undertake the tasks required to assist their care‐recipient to use HMR. In this context, a care‐recipient was a person who was eligible to receive HMR but had not previously received the service. Enhancing participation among caregivers could be challenging because research has demonstrated a lack of willingness to participate among *patients* who have not yet experienced the service.^22^ In addition, caregivers of frail, older patients tend not to use many of those services available to them, and further work is needed to understand how to encourage participation.^23^, ^24^

At present, there is currently no existing theoretical framework which links caregivers' stress with their intentions to participate in health services. Furthermore, there are no quantitative studies that have investigated caregivers' willingness to participate in HMR. A better understanding of the factors that influence caregivers' willingness to participate would provide insights to improving the development and implementation of medication management services. Specifically, descriptions of HMR intended for caregivers could better promote participation in the service. Therefore, the aim of the research was to *develop and test* a model of caregivers' willingness to participate in HMR (for their care‐recipient).

## Methods

The theoretical model for this study was firstly developed then tested in a cross‐sectional survey of caregivers.

### Knowledge Hassles Information Seeking Model (KHISM) development

**Figure 1:**
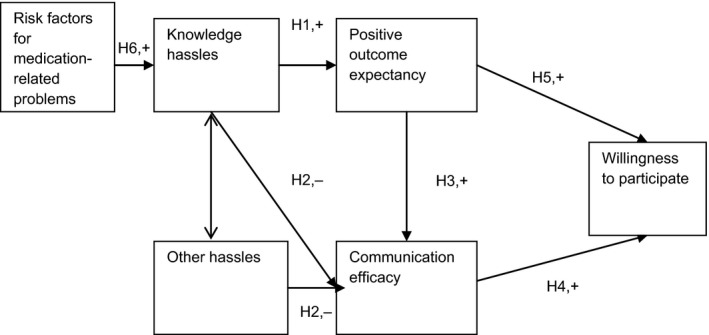
Knowledge Hassles information‐seeking model (KHISM): informal caregivers' willingness to participate in Home Medicines Reviews. + or – refers to the hypothesised direction of influence.

Based on the literature and qualitative studies,^17^, ^25^ several constructs were adapted to the context of the study. The study explored caregivers' willingness to participate in HMR, after being presented with information about the service. It is hypothesized that caregivers' motivations to use HMR are primarily influenced by their expectations of HMR as a medication information source. In other words, their willingness to participate is dependent on medication information seeking. In this context, the Knowledge Hassles Information Seeking Model (KHISM) model creates links between caregiver stress dealing with medication information; expectations about the outcomes and processes involved in HMR; and willingness to participate (Fig. 1).

#### Theoretical foundation

According to cognitive–social theories, whether a person undertakes a particular activity is influ‐enced by their behaviour‐outcome expectancy (*outcome expectancy*) and their self‐efficacy expectancy (*self‐efficacy*). These expectancies are essential components of Bandura's Social Cognitive Theory (SCT),^26^ Rogers' and Maddux's Protection Motivation Theory (PMT)^27^ and Afifi and Weiners' Theory of Motivated Information Management (TMIM).^28^ Of these theories, TMIM most closely suits the present investigation because it deals with information seeking from interpersonal sources.

#### Intention – Willingness to participate

Most of the research conducted with SCT uses *behavioural intention* as the antecedent variable to actual behaviour. In general terms, therefore, our model includes variables related to intention. However, Gibbons *et al*.^29^ argue that willingness to perform a behaviour, that is *intention conditioned on certain premises*, may explain more variation in actual behaviour than intention alone. Willingness may capture irrational and reactive influences.^29^ Therefore, willingness to participate in HMR forms the dependent variable for this model. Based on a qualitative study^30^ and a quantitative study conducted with patients,^31^ willingness to participate was therefore defined as whether respondents would help arrange an HMR for their care‐recipient, whether the care‐recipient's general practitioner (GP) recommend it, and whether they would ask the GP for an HMR if they had concerns about their care‐recipients' medicines.

#### Outcome expectancy

Outcome expectancy deals with an individual's focus on the behavioural outcomes to be derived from information seeking, which in this case is participating in HMR. Outcome expectancy may be further divided into positive expectancy and negative expectancy. This evaluation broadly follows a benefit vs. cost trade‐off. In identifying potential positive outcome expectancy for HMR, the literature suggests that *patients* expect to receive personally relevant medication information that would be reassuring and assist in self‐management.^32^, ^33^, ^34^ Caregivers too had similar expectations in focus group research.^17^, ^35^ Positive outcome expectancy (OE) was therefore defined as a caregiver's beliefs about the effectiveness of an HMR to correct their knowledge deficiency; improve their medication management capability; and to reduce their anxieties about the safety of their care‐recipients' medication regimen. In SCT, positive outcome expectancy increases intentions to act. In patients, positive outcome expectancy (of HMR) was strongly associated with increased willingness to participate in HMR.^31^ While it is possible that negative outcome expectancy – related to the caregivers' potential discomfort of being visited at home – may have a negative effect on their willingness to participate, this category of beliefs has not shown to be influential in patients^31^ and was not therefore investigated in the present model.

#### Self‐efficacy – communication efficacy

Self‐efficacy deals with an individual's perception of their ability to perform the information‐seeking action.^26^ In SCT, self‐efficacy increases intention to act. In the present context, self‐efficacy most closely follows *communication efficacy* in accordance with TMIM.^28^ Here, communication efficacy centres on a caregiver's perception of their confidence to overcome potential barriers in the communication process such as making time for an HMR; organizing it and, if required, asking the patient's doctor to initiate it.^30^ In a study conducted with patients, lack of confidence to perform these tasks decreased willingness to participate in HMR,^31^ and it is likely the same would hold for caregivers.

In SCT, self‐efficacy is thought to influence a person's intention to act both directly and indirectly by increasing outcome expectancy.^26^ Maddux,^36^ Afifi and Weiner,^28^ however, contend that the direction of influence between self‐efficacy and outcome expectancy is reversed. That is, that outcome expectancy influences self‐efficacy. Both sides of the debate offer compelling arguments to support their respective theories; however, there remains an element of uncertainty in the true direction of the causal relationship between these constructs, and the manner in which the controversy could be resolved.^37^ In a cross‐sectional study such as this, it is not possible to determine the direction of influence, and the authors share Maddux, Afifi and Weiners' view that outcome expectancy influences communication efficacy.

#### Daily hassles – antecedent to outcome expectancy and communication efficacy

In the context of health, information seeking is often framed as a coping strategy, which attempts to reduce the cognitive stress and emotional arousal arising from uncertainty.^38^ For example, TMIM,^28^ which adapts constructs from SCT^26^ and PMT,^27^ suggests that an individual's motivation to engage in information seeking arises when an individual is no longer comfortable with their level of uncertainty. In this case, the person experiences negative affect (anxiety) which motivates that person to consider the outcomes expectancy of information seeking.^28^ However, rather than investigating the relationship between outcome expectancy and anxiety (arising from discomfort with uncertainty), this model explores the relationship between outcome expectancy and daily hassles. KHISM proposes that when a person experiences daily hassles, specifically related to knowledge processing, they are motivated to consider outcome expectancy of information seeking.

Daily hassles are the ‘irritating, frustrating, distressing demands that to some degree characterize everyday transactions with the environment’. Experiencing hassles can represent an on‐going and insidious threat to health. For example, the frequency and severity of daily hassles is a better predictor of psychological distress and somatic disease than major life events such as death in a relative, marital changes and serious financial problems.^39^ Past research has shown that experiencing daily hassles with friends, family, environment and life's practicalities is associated with negative affect.^40^ Furthermore, experiencing daily hassles is associated with the tendency to interpret events as threatening and with the tendency to seek out threat‐relevant information (known as ‘monitoring’^41^).^40^

It is known that caregivers experience daily hassles as a result of performing tasks related to managing their care‐recipients' medicines.^42^, ^43^ These daily hassles have also been documented among Mexican caregivers' who speak the Spanish language at home.^44^ Consider the task involved in managing a care‐recipients complex medication regimen. The caregiver needs to understand the processes involved in the procurement and administration of medicines. These processes may require caregivers to negotiate with prescribers, pharmacists, nurses and the care‐recipient. They may need to understand how and when the medicines are taken, monitor for beneficial effects and adverse effects, adjust doses and/or cease medicines. Travis and colleagues have investigated the dimensionality of these daily hassles and have developed the Family Caregiver Medication Administration Hassles (FCMAHS) Scale.^42^ In one setting, the FCMAHS scale had four dimensions: (i) information seeking/information sharing, (ii) safety issues, (iii) scheduling logistics, and (iv) polypharmacy. In another study, factor analysis revealed that data were best represented by six dimensions: (i) initial information seeking, (ii) safety issues, (iii) advanced information acquisition, (iv) scheduling, (v) daily routine, and (vi) prescription filling.^44^

#### Knowledge hassles

This study further extends an understanding of the influence of daily hassles on information seeking by proposing that only specific types of hassles, *knowledge hassles* cause a person to consider the outcomes of information seeking, whereas the other types of hassles will not. Knowledge hassles were defined generally as those daily hassles experienced because of the need to repeatedly process information on a health topic which requires specialized knowledge. KHISM suggests that when a person experiences knowledge hassles, they are likely to consider an interpersonal information source on the health topic particularly relevant and reassuring. In other words as knowledge hassles rise so does positive outcome expectancy.

In this study, the authors compare the effect of *other hassles* with knowledge hassles. In the present context, knowledge hassles are daily stressors experienced by caregivers while dealing with tasks that require knowledge about the safety and effectiveness of the care‐recipients' medicines, whereas the other hassles are medication administration hassles that result from performing tasks which do not require specialized knowledge. Other hassles include hassles with interpersonal interactions between the caregiver and care‐recipient and hassles with maintaining the logistics of supply. In seeking to separate knowledge hassles from other hassles, eight items from FCMAH scale were adapted. The authors chose not to use the whole FCMAH scale to produce a reasonably parsimonious questionnaire. This was also carried out to reduce the cognitive load on the older respondents. For the purpose of this study, four items were selected to represent knowledge hassles: recognizing adverse effects; knowing whether the medicine is effective; knowing why the medicine is used; and knowing what questions to ask the doctor. Four items were selected to represent other hassles: scheduling medicines into the daily routine; sharing responsibility with the care‐recipient; arguing with care‐recipient about when to take medicines; and giving medicines on time. Experiencing other hassles would not necessarily have any influence over positive outcome expectancy because seeking information (about medicines) would not resolve these stressors.

In seeking to understand how experiencing hassles may influence communication efficacy, it is clear that there is a close association between experiencing hassles and anxiety.^40^ There is also a close relationship between anxiety and low self‐efficacy.^26^ It is possible that experiencing hassles may therefore have a detrimental effect on the self‐confidence required to perform the communication tasks associated with information seeking. Whereas only knowledge hassles should influence positive outcome expectancy, experiencing *any hassles* may decrease communication efficacy.

#### Situational factors that may influence knowledge hassles

While daily hassles are persistent daily stressors, it is expected that the level of hassles would fluctuate according to certain situations. Knowledge hassles would fluctuate according to a person's current perception of the specialized knowledge demanded of them at the time. Knowledge hassles would be more stressful during extended episodes of uncertainty. There are three situations in the present context, which would be associated with extended periods of uncertainty and potentially heightened knowledge hassles; (i) while a caregiver adjusts to the demands of changes to the care‐recipient's medication regimen. This could occur quite frequently for care‐recipients who use multiple medicines for chronic diseases, (ii) while a caregiver adjusts to changes in the care‐recipients' overall health after hospitalization. It should also be noted that medication regimens are changed quite frequently during hospitalization and extensive changes increase the risk of further changes in the regimen after discharge,^45^ and (iii) a caregiver would perceive extra demand on knowledge if they were required to implement the instructions of multiple regular prescribers, which could at times be conflicting.^46^ Each of the situations mentioned above: (i) having a change in the regimen within the past 3 months, (ii) having been hospitalized within the past month, and (iii) having multiple regular prescribers, place an individual at increased risk of experiencing medication‐related problems.^47^, ^48^ These risk factors may also be used to identify patients who may benefit from HMR.^13^

The KHISM model tests the following hypotheses.

- Knowledge hassles (but not other hassles) would increase positive outcome expectancy (H1).
- Knowledge hassles and other hassles would decrease communication efficacy (H2).
- Positive outcome expectancy would increase communication efficacy (H3).
- Communication efficacy would increase willingness to participate (H4).
- Positive outcome expectancy would increase willingness to participate (H5).
- Knowledge hassles would be heightened in the presence of three risk factors for medication‐related problems: (i) having a change in the regimen within the past 3 months, (ii) having been hospitalized within the past month, and (iii) having multiple regular prescribers (H6).

### Model testing

#### Respondents

During February and March 2009, respondents were recruited through mail‐out to 2350 members of a caregiver interest group, based in Sydney, Australia; Carers (NSW). No incentives were offered to respondents or Carers (NSW). Approval for the project was given by the University of Sydney Human Ethics Committee.

##### Inclusion criteria

The study included informal caregivers of adult persons who had not previously experienced HMR but were eligible because they were taking more than five medicines daily or more than 12 doses daily.^13^ Informal caregivers were defined as those who were not paid to provide care, other than receiving government allowances and who provided a certain level of care. Therefore, the study included caregivers who *sometimes, often* or *always* were involved in at least one of the following four medication‐related caring duties for their care‐recipient: Purchase, order or collect his/her medicines; organize how and when he/she takes the medicines; give him/her a dose; or make decisions to increase or decrease a dose, or not take a dose, or discontinue the medicine altogether.^42^

#### Questionnaire

##### Caregiver and care‐recipient characteristics

The demographic information collected in the questionnaires included the caregivers' and care‐recipients' gender, age, education and location by postcode. Data were also collected on the relationship status between caregiver and care‐recipient, the level of care provided and whether the caregiver was paid to provide care. To determine whether the care‐recipient had risk factors for medication‐related problems, respondents were asked whether (i) there had been a change in the care‐recipients' medicines or doses prescribed within the past 3 months, (ii) whether the care‐recipient had been discharged from hospital within the previous month, and (iii) whether the care‐recipient had multiple regular prescribers. These questions had a response format of *yes*,* no* and *not sure*.

##### Psychosocial measures

Daily hassles. As explained in the model development section, four items were used to measure each of the knowledge hassles (KH) and other hassles (OH) constructs. Respondents were asked to rate each task on a scale from 0 = *no hassle at all* to 5 = *the worst of all hassles* as to how much of a hassle it is to help manage the care‐recipient's medicines.

All other psychosocial measures were adapted from a questionnaire conducted with patients.^31^ Each of the Likert scales had a response format of 1 = strongly disagree to 5 = strongly agree. The questions were modified such that caregivers' beliefs, thoughts and feelings were examined in relation to caregiving. For example: If I had a Home Medicines Review, managing my medicines would be easier was reconstructed to: If (the person I care for) had a Home Medicines Review, managing (the person I care for)'s medicines would be easier.

Outcome expectancy (OE). Respondents provided their level of agreement with six items on a 5‐point Likert scale. The scale has shown to have good construct reliability (0.94) among patients.^31^

Communication efficacy (CE). Respondents provided their level of agreement with four items on a 5–point Likert scale. Note that these items were reverse coded prior to further analysis. The scale has shown to have acceptable construct reliability (0.75) among patients.^31^

##### Dependent variable

Willingness to participate (W). Respondents provided their level of agreement with two items on a 5–point Likert scale. The scale has shown to have acceptable construct reliability (0.71) among patients.^31^

The questionnaire was examined for face validity by a panel of seven expert community pharmacists, consultant pharmacists and pharmacy academics. A short explanation of the service was provided which was adapted from an Australian Government consumer brochure (Appendix).^49^

#### Analysis

PASW version 18.0.03 (SPSS Inc., Chicago, IL, USA, www.spss.com) was used for descriptive statistical analyses, multiple regression and exploratory factor analyses (EFA). Principal components analysis was used with oblimin rotation because the scales were expected to be correlated. Confirmatory factor analysis (CFA) and structural equation modelling (SEM) were performed with EQS 6.1 build 97 (Multivariate Software Inc., Encino, CA, USA, www.mvsoft.com). Hypothesis testing was performed by multiple regression analysis and with SEM. Evidence of data non‐normality in willingness to participate required that structural equation parameter estimates were made using maximum likelihood estimation with robust errors (which is used within the EQS program). All eight items contained within the two hassles scales were subject to an initial EFA to confirm that there were two dimensions. Each of the other multi‐item measurement scales was also subject to initial EFA to determine dimensionality and to detect items with low communality (<0.5).^50^ Following this procedure, CFA was performed in the presence of willingness to participate (W).

#### Confirmatory factor analysis

Convergent validity of the constructs was assessed by inspection of the results from CFA. Standardized factor loadings should exceed 0.50 with statistical significance, to demonstrate high convergence on a common point.^50^ In addition, the average variance extracted should equal or exceed 50%.^50^ The reliability of the constructs was computed using the formula suggested by Fornell and Larcker.^51^ The construct reliability values equal to or greater than 0.7 indicate that the construct of the model is reliable, although coefficients of between 0.5 and 0.8 may be considered acceptable during preliminary investigations.^50^ Discriminant validity was assessed through the use of variance‐extracted test.^51^ Constructs were evaluated by comparing the variance‐extracted estimates for two factors with the square of the correlation between the two factors. Discriminant validity is demonstrated if both variance‐extracted estimates are greater than the squared correlation. Measurement errors were fixed to (1‐reliability) X variance.^52^

#### Structural equation model

Using the method of Westlund,^53^ it was calculated that the minimum sample size for an appropriate indicator to latent ratio, with five latent constructs and 19 indicators, to be 112. Therefore, the study had sufficient power to perform CFA. Using the method of Westlund,^53^ it was estimated that a sample size of more than 344 was required to detect a minimum effect size of 0.20 with a power of 0.8 and *P* < 0.05. Using the same method, *post hoc*, it was estimated that the minimum effect size that could reliably be determined with the sample of 297 (the number of respondents with a complete data set) and the same power and significance settings was 0.22.

#### Multiple regression analysis

A stepwise linear regression analysis was used to test the influence of each of the three situational factors on knowledge hassles. For this procedure, the dependent variable was the summated factor‐based score, which was calculated by summing the responses to each of the four knowledge hassle items. The possible range for the factor score was 0–20. Prior to performing the regression, the skewness and kurtosis of the summated factor score was calculated. Independent variables were caregiver characteristics, gender, age, education level, and the three risk factors for medication‐related problems. The reference category for the risk factors was ‘no’ or ‘not sure’.

## Results

Questionnaires were received from 600 respondents and of these, 324 met the inclusion criteria. This provided a 14.4% effective response rate. Descriptive statistics of the sample are provided in Table 1.

**Table 1:** Descriptive statistics

Demographic characteristics^a^	Caregiver	Care‐recipient
	Mean (SD), range	Mean (SD), range
Age	64.6 (11.8), 27–88	67.7 (18.2), 18–98
	*n* (%)	*n* (%)
Gender		
Male	87 (26.9)	167 (52.2)
Female	236 (73.1)	153 (47.8)
Relationship		
Spouse		207 (64.3)
Other family relationship		101 (31.4)
Unrelated		14 (4.3)
Location		
Metropolitan	272 (84.0)	273 (84.3)
Rural or remote	52 (16.0)	51 (15.7)
Education level^b^		
Year 10 or below	107 (33.8)	147 (45.9)
Year 12 or equivalent	100 (31.7)	113 (36.5)
University	108 (34.3)	50 (16.1)
Medication risk factors^c^		
Change in the medication regimen within the previous 3 months		129 (41.0)
Discharged from hospital within the previous month		44 (13.8)
Multiple regular prescribers		113 (35.9)
Frequency of care provided with medicine tasks^d^		
Purchase, order or collect his/her medicines	315 (97.2)	
Organize how and when he/she takes the medicines	273 (84.8)	
Give him/her a dose	235 (73.0)	
Make decisions to increase or decrease a dose, or not take a dose, or discontinue the medicine altogether	56 (17.3)	

a Refers to valid responses only, so that the sum of responses may not add up to the total.

b Within Australia, the two categories of high school education level; year 10 and year 12, indicate eleven and 13 years of formal school education, respectively.

c Respondents were divided into two groups, those who answered ‘yes’, or those who answered ‘no’ or ‘don't know’. The numbers and proportions refer to those who answered ‘yes’.

d Refers to the proportion who responded ‘sometimes’, ‘often’ or ‘always’ to the level of care provided to the care‐recipient.

### Psychosocial measures

Means and standard deviations for the psychosocial measures from each group are presented in Table 2. The following provides some descriptive statistics of the belief measures and the results of EFA for each construct.

**Table 2:** Descriptive statistics of psychosocial measures

Construct	Item code	Item	Mean (SD)	*n*
Hassles^a^				
Knowledge hassles	KH1	Recognizing adverse effects	1.66 (1.54)	322
	KH2	Knowing whether the medicine is effective	1.66 (1.56)	323
	KH3	Knowing why the medicine is used	0.88 (1.32)	322
	KH4	Knowing what questions to ask the doctor	1.25 (1.42)	323
Other hassles	OH1	Scheduling the medicines into the daily routine	0.71 (1.17)	322
	OH2	Arguing with care‐recipient about when to take medicines	0.95 (1.37)	319
	OH3	Sharing responsibility with the care‐recipient	0.91 (1.39)	323
	OH4	Giving medicines on time	0.87 (1.25)	322
Outcome expectancy^b^	OE1	Ease with managing the medicines	3.01 (1.01)	318
	OE2	Fewer concerns about long‐term side‐effects	3.25 (1.09)	320
	OE3	Fewer concerns about drug interactions	3.32 (1.11)	318
	OE4	More confident the medicines are helping	3.29 (1.08)	320
	OE5	Understand more about the medicines	3.40 (1.10)	317
	OE6	Assist care‐recipient to live at home independently	2.80 (1.07)	316
Communication efficacy^b^	CE1	Difficulty arranging (reverse score provided)	3.62 (0.97)	318
	CE2	No time (reverse score provided)	4.00 (0.95)	319
	CE3	Asking for Home Medicines Review (HMR) indicates that I have no confidence in GP (reverse score provided)	3.66 (0.97)	319
	CE4	Difficulty asking GP (reverse score provided)	3.76 (0.96)	319
Willingness to participate^b^	W1	Willing to help arrange an HMR if suggested by the GP	3.96 (0.80)	318
	W2	Willing to ask the GP for an HMR if having concerns about medicines	3.88 (0.85)	319

a Responses varied from 0 (no hassle at all) to 5 (the worst of all hassles).

b Responses varied from 1 (strongly disagree) to 5 (strongly agree).

#### Daily hassles

Overall, respondents reported experiencing low levels of daily hassles related to managing their care‐recipients' medicines. Yet, a minority of respondents (*n *= 60, 18.5%) recorded 0 (no hassles at all) to all eight daily hassles items. The median score for three of the four knowledge hassles (KH) items was 1, whereas the median was 0 for the item which dealt with hassles related to knowing why a medicine is being given. The median score for each of the four other hassles (OH) items was 0. Following EFA, eight items loaded onto two factors with eigenvalues above 1 which explained 65.3% of the variation. All items had communalities above 0.5, and all items loaded onto the expected factors. The factor loadings ranged between 0.63 and 0.93, and there were no cross‐loadings above 0.3.

#### Outcome expectancy

For most variables, the median score was 3, the neutral response. This indicates that overall, respondents were not convinced that an HMR would provide these positive outcomes. All six items loaded onto the one factor with eigenvalues above 1 which explained 76.4% of the variance. All items had communalities above 0.5. The factor loadings ranged between 0.78 and 0.92.

#### Communication efficacy

Following reverse coding, the median score for each variable was four, indicating overall high levels of communication efficacy. All four items loaded onto the one factor with eigenvalues above 1 which explained 48.5% of the variance. Two items had communalities below 0.5, being 0.47 and 0.44 for CE3 and CE4, respectively. These items were retained to provide a multi‐item scale for the study. Factor loadings ranged between 0.67 and 0.73.

#### Willingness to participate

The median score for each of these items was four, indicating that overall, respondents were willing to participate in HMR if suggested by the GP and willing to ask the GP if they had concerns about the care‐recipients medicines. This two item scale was not subjected to EFA.

### Confirmatory factor analysis

Missing data analysis revealed that the overall level of missing data was small (<5% for all variables), and only 27 cases were excluded. Examination of the correlation matrix revealed that no relationships were above 0.90; therefore, multicollinearity was not considered problematic.

The CFA fit statistics indicated that the measurement model was a reasonable fit for the data. Apart from the significant Satorra‐Bentler scaled chi‐square = 320, d.f. = 160, *P* < 0.001, model fit indices were good for the measurement model. Model fit statistics: CFI = 0.94, TLI = 0.93, RMSEA = 0.058 (90% confidence interval = 0.049, 0.067). Standardized and unstandardized factor loadings, construct reliabilities and average variances extracted are presented in Table 3. The variances of the indicator variables loading onto each latent construct were significant. For each of the constructs, with the exception of communication efficacy, all of the factor loadings were greater than or equal to 0.48, and average variances extracted were greater than or equal to 50%. For these scales, the minimum construct reliability estimate was 0.71. There was some question, however, about the reliability of the CE scale. Two of the items had relatively low factor loadings of 0.42 for CE3 and 0.40 for CE4. In addition, construct reliability (0.62) and average variance extracted (30%) were below the limit of acceptability by the standards preset. The performance of the scale could not be improved by deleting either item; therefore, these items were retained to provide a multi‐item scale for this study. Discriminant validity between each of the constructs was demonstrated, as for each pair of constructs both average variances extracted estimates were greater than the squared correlation.

**Table 3:** Confirmatory factor analysis (*N *=* *324)

Item	Standardized regression weights	Unstandardized regression weights (URW)	Robust SE of URW	Construct reliability	Average variance extracted (%)
KH1	0.73	1.12	0.06	0.85	59
KH2	0.57	1.00	0.08		
KH3	0.63	0.71	0.08		
KH4	0.82	1.00	0.00		
OH1	0.73	1.00	0.00	0.79	50
OH2	0.53	0.84	0.14		
OH3	0.46	1.07	0.13		
OH4	0.68	1.18	0.11		
OE1	0.79	0.80	0.04	0.94	72
OE2	0.92	1.00	0.00		
OE3	0.91	1.01	0.03		
OE4	0.89	0.95	0.03		
OE5	0.84	0.92	0.04		
OE6	0.71	0.76	0.05		
CE1	0.62	1.00	0.00	0.62	30
CE2	0.69	1.12	0.19		
CE3	0.42	0.70	0.16		
CE4	0.40	0.65	0.15		
W1	0.81	1.00	0.00	0.71	56
W2	0.67	0.91	0.11		

KH, knowledge hassles; OH, other hassles; OE, outcome expectancy; CE, communication efficacy; W, willingness to participate.

### Structural equation model

**Figure 2:**
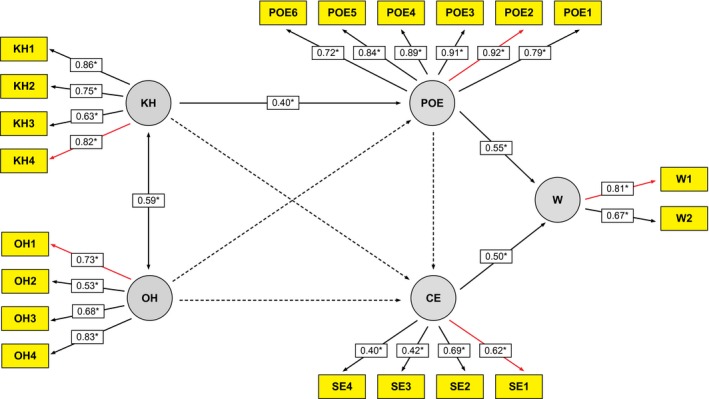
Structural equation model. Solid lines indicates significant correlation and regression coefficients (*P < 0.05). The dashed lines indicate no significant relationship. *R* ^2^ = 0.54. KH = Knowledge hassles, OH = Other hassles, POE = Positive outcome expectancy, CE = Communication efficacy, W = Willingness to participate.

The SEM fit statistics indicated that the measurement model was a reasonable fit for the data. Apart from the significant Satorra‐Bentler scaled chi‐square = 321, d.f. = 162, *P* < 0.001, model fit indices were good for the measurement model. Model fit statistics: CFI = 0.94, TLI = 0.93, RMSEA = 0.058 (90% confidence interval = 0.048, 0.067). The model predicted 54% of the variation in willingness (W), 18% of the variation in outcome expectancy (OE) and just 3% of the variation in communication efficacy. Figure 2 provides the results of hypothesis testing. Outcome expectancy (OE) (β = 0.55, *P* < 0.05) and communication efficacy (β = 0.50, *P* < 0.05) had strongly positive effects on willingness (W). Knowledge hassles (KH) had a moderate effect (β = 0.40, *P* < 0.05) on outcome expectancy (OE) but no significant effect on communication efficacy (CE). Knowledge hassles (KH) had weak indirect effects on willingness (W) (β = 0.19, *P* < 0.05). Other hassles (OH) were correlated with knowledge hassles (KH) (*r* = 0.59, *P* < 0.05) but had no significant effect on other variables in the model.

### Multiple regression

The summated knowledge hassle factor score had a skewness of 0.64 and kurtosis of −0.47 and was therefore determined to be an appropriate dependent variable for linear regression. The stepwise regression resulted in a model with the following statistics; adjusted *R*‐squared = 0.082, *F* = 14.16, *P* < 0.001. There were two significant predictor variables (*P* < 0.05); having a recent change in the medication regimen (β = 0.21, *P* < 0.001) and age of the respondent (β = −0.16, *P* = 0.006).

## Discussion

This study demonstrates that a majority of informal caregivers experience a specific type of daily hassles as a consequence of their role as medication managers. The authors coined the phrase ‘knowledge hassles’ to describe the hassles experienced when dealing with the specialized knowledge of their care‐recipients' medicines' effects and side‐effects. While the overall levels of these hassles were not high, they were clearly influential. As predicted, the higher the level of these hassles, the more *personally* beneficial for them a caregiver would find HMR and the more willing they would be to arrange an HMR for their care‐recipient. This effect was expected by the researchers because caregivers' positive outcome expectancies of HMR centre on receiving information about medication issues, reassurance about medication‐related concerns and improvement of medication management capability. These expectancies cover the same topics as patients and have the same motivating effect on willingness to participate.^31^ Similar to the experience with patients, caregivers' expectancies were fairly neutral, and they were overall unconvinced that HMR would provide these benefits.^22^, ^31^These findings align with other research which suggests that many users of pharmacy services do not expect that pharmacists would provide modern pharmaceutical care services.^54^ Because these expectancies are relatively low, there appears to be significant scope for increasing caregiver demand for medication management services.

Alternatively, experiencing ‘other hassles’, the daily hassles resulting from tasks which do not require knowledge of medicines, was not found to be influential. This is the first time that a relationship has been drawn between the *specific* feelings of being stressed about processing information on a health topic; and a willingness to seek information about that topic. These findings have important implications for pharmacotherapy and for the development of theory of information‐seeking behaviour. Further research into knowledge hassles is warranted. If confirmed in other contexts, the assessment of knowledge hassles could be a useful tool in health provider – client communication.

It is noteworthy that it was the oldest of the caregivers who experienced the least knowledge hassles. This is consistent with an observed decline in stress levels with age among Australian caregivers.^55^ Such changes may be explained by socioemotional selectivity theory.^56^ It is believed that ‘age‐related constraints on time horizons’ are associated with motivational changes. These changes cause older persons to increasingly focus on positive over negative emotions.^56^ It is possible that older caregivers' declining experience of daily knowledge hassles may prevent them from focussing on the outcomes of participating in health information services.

### Limitations

The main limitation of the study was the potential for bias within the particular group of respondents which may limit the generalizablity of the results. These caregivers were recruited because they belonged to a support group, Carers NSW. One of the core goals of this support group is to ‘Develop, promote and distribute information, resources and publications to carers’. Therefore, the respondents recruited for this study may be more likely than others to seek out information about caregiving tasks. Further studies could be conducted among different populations using the measurement scales developed within this study to examine the relationships between the key variables. Another limitation was the relatively low effective response rate (14.4%). This resulted from an overall poor response 600/2350 (25.5%) and the strict inclusion criteria.

Another limitation to the study was that the measurement scale for communication efficacy was not as reliable as had been hoped. It is possible that the construct, communication efficacy is multidimensional and that the present study used an inadequate number of indicators (four) to tap the dimensionality of this latent construct. Despite the modest reliability of the scale, communication efficacy appeared to have a strong influence over willingness to participate. Future studies could use more indicators or alternative indicators to determine the dimensionality of this influential construct.

The measurement scales used for the hassles constructs used only selected items from Travis *et al*.'s FCMAHS scale.^9^ Ideally, the study should be replicated with the complete scale.

## Conclusions

This study highlights that some caregivers experience quite a degree of stress dealing with medication information. As expected, this stress is heightened when the medication regimen is changed. Services and support should be provided to these caregivers to assist them in their important and unpaid role as the communities' hands‐on medication managers. Home Medicines Reviews is one avenue for supporting the information needs of caregivers. Building expectations of HMR as an information resource among informal caregivers would likely increase overall consumer demand for this service and may ease the stress and burden of caregiving. General practitioners who sense that informal caregivers seem stressed about medication information will most likely find them quite receptive to suggestions to have HMR, particularly after the medication regimen has changed.

## Conflict of interest

The authors declare no conflict of interest.

## Sources of funding

This project was funded by the Australian Government Department of Health and Ageing as part of the Fourth Community Pharmacy Agreement Research & Development Program managed by the Pharmacy Guild of Australia.

## Appendix

Description of HMR provided to eligible non‐recipients, adapted from an Australian Government consumer brochure.^49^
A Home Medicines Review, a free service funded by Medicare, provided jointly by your General Practitioner (GP) and pharmacist, is particularly useful for people who take multiple medicines each day, or who have recently spent time in hospital or who are concerned or uncertain about their medicines. After being referred by a GP, the pharmacist usually visits the patient in their own home at a mutually agreed time. The pharmacist will look at all medicines that the patient has, discuss any difficulties or concerns the patient may have with using their medicines and write a report to the GP. The GP will then discuss the results of the Home Medicine Review with the patient. Home Medicine Reviews help patients and carers to understand better how to use their medicines.
